# A Natural Small Molecule Harmine Inhibits Angiogenesis and Suppresses Tumour Growth through Activation of p53 in Endothelial Cells

**DOI:** 10.1371/journal.pone.0052162

**Published:** 2012-12-27

**Authors:** Fujun Dai, Yihua Chen, Yajuan Song, Li Huang, Dong Zhai, Yanmin Dong, Li Lai, Tao Zhang, Dali Li, Xiufeng Pang, Mingyao Liu, Zhengfang Yi

**Affiliations:** 1 Shanghai Key Laboratory of Regulatory Biology, Institute of Biomedical Sciences and School of Life Sciences, East China Normal University, Shanghai, China; 2 Center for Cancer and Stem Cell Biology, Institute of Biosciences and Technology and Department of Molecular and Cellular Medicine, Texas A&M University Health Science Center, Houston, Texas, United States of America; Rush University Medical Center, United States of America

## Abstract

Activation of p53 effectively inhibits tumor angiogenesis that is necessary for tumor growth and metastasis. Reactivation of the p53 by small molecules has emerged as a promising new strategy for cancer therapy. Several classes of small-molecules that activate the p53 pathway have been discovered using various approaches. Here, we identified harmine (β-carboline alkaloid) as a novel activator of p53 signaling involved in inhibition of angiogenesis and tumor growth. Harmine induced p53 phosphorylation and disrupted the p53-MDM2 interaction. Harmine also prevented p53 degradation in the presence of cycloheximide and activated nuclear accumulation of p53 followed by increasing its transcriptional activity in endothelial cells. Moreover, harmine not only induced endothelial cell cycle arrest and apoptosis, but also suppressed endothelial cell migration and tube formation as well as induction of neovascularity in a mouse corneal micropocket assay. Finally, harmine inhibited tumor growth by reducing tumor angiogenesis, as demonstrated by a xenograft tumor model. Our results suggested a novel mechanism and bioactivity of harmine, which inhibited tumor growth by activating the p53 signaling pathway and blocking angiogenesis in endothelial cells.

## Introduction

The tumor suppressor p53 plays a key role in the regulation of cell cycle, apoptosis, DNA repair, and senescence [1] in response to diverse stress stimuli, including DNA damage, oncogene activation and hypoxia [2]. p53 acts as a transcriptional factor and activates various genes to exert specific functions involved in tumor development. Murine double minute 2 (MDM2), the main regulator of p53, inhibits the function of p53 through direct interaction [3].

In addition to the direct effect of targeting tumor cells, there are accumulating evidences that show activation of p53 may also effectively inhibit angiogenesis, which is one of the most important hallmarks in the cancer development [4], and is critical for tumor growth and metastasis [5]. Overexpression of p53 inhibits angiogenesis by up-regulation of its downstream target genes such as thrombospondin 1 (TSP-1) [6] and brain-specific angiogenesis inhibitor 1 (Bai1) [7]. Therefore, the p53 activation is also fundamentally involved in angiogenic processes [5].

Because of the high potential of p53 to elicit apoptosis or growth arrest in cells, pharmacological reactivation of the p53 tumor suppressor is a promising strategy for anti-cancer therapy [8]. Recently, proposed model suggested that p53 activation *in vivo* includes three major steps: (1) p53 stabilization, (2) release from MDM2 (i.e. antirepression) [9], and (3) promoter-specific activation [10]. Previous reports showed that some small compounds induces cancer cell cycle arrest and apoptosis through restoration of p53 pathway [2], [11] and some other small molecules such as Inauhzin were identified that induced the level and activity of p53 consequently and effectively repressed the growth of xenograft tumours [12]. Indeed, several p53-reactivating compounds are currently being tested in clinical trials, including mutant p53-reactivating PRIMA-1 analog APR-246 [8].

Harmine, a small-molecule β-carboline alkaloid, is a naturally occurring compound in some plant species [13]. Previous research has shown that harmine plays some roles in anti-cancer treatments [14], as well as possesses anti-leishmanial properties [15] and an anti-viral effect [16] via multiple signaling pathways such as kinase [17] and mitochondrial signaling pathways [18]. However, there are no evidences that show harmine or its analogues exert their bioactivities via the p53 signaling pathway.

In the present study, we identified harmine as a novel activator of the p53 pathway and characterized its anti-angiogenic and anti-tumor effects via p53 signaling pathway in endothelial cells.

## Materials and Methods

### Cell Lines, Animals and Regents

Human umbilical vein endothelial cells (HUVECs) were purchased from Sciencell Research Laboratories (Beijing, China). Human A549 lung cancer cells provided by ATCC were cultured in RPMI 1640 medium. Mice were obtained from National Rodent Laboratory Animal Resources (Shanghai, China). All animal procedures were approved by the institutional Animal Ethics Committee of East China Normal University. Vascular endothelial growth factor (VEGF) and growth factor-reduced Matrigel were purchased from R&D Biosciences (San Diego, CA). p53-siRNA and negative control oligonucleotides were purchased from GenePharma (Shanghai, China). Harmine (≥98% purity) was obtained from Sigma-Aldrich).

### Western Blotting and Co-immunoprecipitation

Harvested cells were lysed in RIPA buffer containing protease/phosphatase inhibitors (Roche). Lysates were combined with sample loading buffer and heated at 100°C. Cytoplasmic and nuclear extractions were performed. For the co-immunoprecipitation assay, whole cell lysates or fractionated samples were incubated with specific antibodies overnight for precipitation and then incubated with protein A/G-Sepharose beads (GE Healthcare Bio-Sciences). Protein samples were eluted in sample buffer and subjected to SDS-polyacrylamide gel electrophoresis. Antibodies used were as follows: anti-p53, anti-MDM2 and anti-TSP1 purchased from Santa Cruz Biotechnology (Santa Cruz, CA), and anti-β actin, anti-phospho-p53 (ser-37), anti-phospho-p53 (ser-20), anti-phospho-p53 (ser-15), anti-p21, anti-cyclin B1, anti-cyclin E, anti-cyclin D1, anti-cyclin A, anti-CDC2, anti-CDK2, anti-Poly (ADP-ribose) polymerase (PARP), anti-Bcl2 and anti-survivin purchased from Cell Signaling Technology (Boston, MA). Antibody of anti-γH2AX was from Abcam Inc. (Cambrige, MA).

### ProteOn XPR36 Protein Interaction Array

Measurement of binding affinities between harmine and MDM2 was performed as described elsewhere [19] with some modification. Briefly, a GLH chip was used in this assay. glutathione S-transferase (GST) and GST-MDM2 were dissolved in 10 mM NaAc and immobilized onto separate erect channels of the sensor chip by general amine coupling. MDM2-GST was immobilized to around 17400 RUs. After baselines were stable, harmine was dissolved in PBS-T buffer flowing through the chip horizontally. Data were analyzed with ProteOn manager™ software using the Langmuir model (A+B ↔ AB) for kinetic data fitting.

### Immunofluorescence Staining

As described in a previous report [20], Harmine-treated HUVECs were fixed using 4% formaldehyde and washed with phosphate buffered saline (PBS). Fixed cells were treated with 0.2% Triton-X 100. After blocking in 0.5% bovine serum albumin, cells were incubated with the primary antibody at room temperature, followed by incubation with the secondary antibody. Then, 4′,6-diamidino-2-phenylindole (DAPI) was added to samples, followed by incubation while protected from light. Images were recorded by confocal microscopy (Leica).

### Real-Time RT-PCR

Total RNA was extracted from cells using Trizol reagent, and then cDNA was synthesized. Real-time PCR was performed using MxPro-Mx3005P software and a STRATAGENE Real-Time PCR instrument. Primers used were as follows: Bai1, forward 5′-ACTCATCCTGCGACGGTGTG-3′ and reverse 5′-TCCCTCAGGT CCTTCATGCG-3′; p21, forward 5′-CTC TCAGGGTCGAAAACGG-3′ and reverse 5′-GATGTAGAGCGGGCCTTT G-3′; survivin, forward 5′-GCGCTTTCCT TTCTGTCAAG-3′ and reverse 5′-TAAGA CATTGCTAAGGGGCC-3′; TSP-1, forward 5′-TATGCTGGTGGTAGACTAGG-3′ and reverse 5′-TCTAGGAG TCCACACTGATG-3′; CDC2, forward 5′-CTAGCATCCCATGTCAAAAAC-3′ and reverse 5′-ACGAAGTACAGCTGAA GTTTG-3′; CDK2, forward 5′-GCCAGGA GTTACTTCTATGC-3′ and reverse 5′-GTCACATCCTGGAAG AAAGG-3′; cylinB1, forward 5′-GACTGTCAAGAACAAGTATGC-3′ and reverse 5′-CAGTAGGAA GTAACCACATTC-3′; P53, forward 5′-TGTTCCGAGAGCTGAATGAG-3′ and reverse 5′-GCAAGCAAGGGTTCAAAGAC-3′. Expression levels of genes were relative to expression of the housekeeping gene β-actin.

### Cell cycle, Apoptosis and Proliferation Analyses

For cell cycle analysis, cells were harvested and washed with PBS. Then, collected cells were re-suspended in 70% cold ethanol and fixed overnight at 4°C. After centrifugation, cells were re-suspended with PBS containing RNase and incubated at 37°C for 30 minutes. Cells were then stained with PI and analyzed by flow cytometry. Hamine-induced apoptosis of HUVECs or HUVECs transfected with p53 siRNA was determined was quantified by an annexin V-FITC/PI dual staining assay. Briefly, cells were collected, centrifuged and re-suspended with PBS. After centrifugation, cells were re-suspended with binding buffer and incubated with annexin V and PI. Analysis was performed by flow cytometry. The effect of harmine on the proliferation of HUVECs was assessed by an MTS assay according to methods described elsewhere [21]. HUVECs or human A549 lung cancer cells were seeded in 96-well plates (5000 cells/well). After culture for 12 hours, cells were treated with various concentrations of harmine for 48 hours. Then, an AQueous One solution (Promega, Madison, WI) was added, and absorbances were measured with a microplate reader (SpectraMax 190; Molecular Devices).

### P53 Silencing Using p53 siRNA

HUVECs were seeded in 6-well plate and transfected with p53 siRNA the next day. The effect of siRNA for p53 silencing was examined by Real-Time RT-PCR and western blot after the transfection for 36 hours and 72 hours respectively. After transfection for 16 hours, cells were treated with various concentrations of harmine for 48 hours.

### Comet Assay

Alkaline comet assays were performed according to the previous study [22] with some modifications. In brief, HUVECs were treated with various concentrations of harmine for 48 hours, and then harvested. About 5 × 10^4^ cells treated with harmine or untreated in 40 µl of phosphate-buffered saline mixed with an equal amount (40 µl) of 1% lower melting agarose were pipetted onto the slides that were covered with 100 µl of 0.5% normal molten agarose at 60°C. The cell suspension spread using a coverslip, and the slides were maintained at 4°C for 15 min to allow the gel to solidify. After removal of the coverslip, the slides were immersed in freshly prepared cold lysis solution (2.5 M NaCl, 100 mM Na2EDTA, 10 mM Tris, pH 10.0, 1% sodium sarcosinate) with 1% Triton X-100 at 4°C for 1 hour. Electrophoresis was carried out in a horizontal gel electrophoresis tank filled with fresh electrophoresis solution (1 mM Na2EDTA, 300 mM NaOH, pH 13.0) for 20 min (300 mA at 10 V) 4°C. Slides were rinsed twice in Tris buffer (0.4 M Tris, pH 7.5) for 15 min (neutralizing the excess alkali) and stained with 75µl of propidium iodide (5 µg/ml) for 30 min. The slides were then examined using a Leica fluorescence microscope under × 200 magnification.

### Cell Migration and Tube Formation Assays

HUVECs were cultured in 6 well plates precoated with 1% gelatin (Sigma-Aldrich) and grown to confluency. After the treatment of mitomycin C for 2 hours, the cells were scratched with a pipette tip followed by the addition of harmine. Images of wound area were recorded after 8 hours, and the number of migrated cells was counted. Another migration test of HUVECs was assessed by a modified transwell assay as described elsewhere [23]. As for 549 lung cancer cells migration assay, cells were seeded in 6 well plates and grown to confluency. After the starvation overnight, the following steps were described as above. After the treatment for 12 hours, the number of migrated cells was counted. During the migration assays, we did not find the phenomenon of apoptosis. For the tube formation assay, 2×10^4^ cells per well were seeded in pre-cooled 96 well plates coated with Matrigel [24] and containing medium with or without harmine, followed by incubation at 37°C with 5% CO_2_. After 12 hours, tube structures in eight randomly chosen fields were photographed and counted by scoring the enclosed networks.

### *Ex vivo* and *In vivo* Angiogenesis Assays

A rat aortic ring assay was performed as described elsewhere [25] with some modification. Briefly, thoracic aortas were sliced into rings of 1–1.5 mm in circumference. VEGF (200 ng) with or without harmine in serum-free MCDB131 medium was added to aortic rings coated with Matrigel. Medium was exchanged every 2 days. On day 7, microvessel outgrowths were photographed and analyzed. A mouse corneal micropocket assay was performed as described elsewhere [26] with some modification. Briefly, slow-release pellets (0.35 × 0.35 mm) containing 200 ng VEGF with or without 5 µg harmine were prepared with a sucrose octasulfate-aluminum complex and poly-HEMA. A corneal micropocket was created in the eye of a 4-5-week-old C57BL/6 mouse with a modified needle, and pellets were implanted into these micropockets. Chlortetracycline hydrochloride ophthalmic ointment was applied to each operated eye to prevent infection. After 7 days, the vessel length and clock hours of new blood vessels were examined under a stereomicroscope and recorded. The area of neovasculature was calculated according to the formula: Area (mm^2^)  = 0.2 × π × VL (mm) × CN (mm), where VL is the maximal vessel length extended from the limbal vasculature toward the pellet and CN is the clock hours of neovascularization, 1 clock hour equals 30 degrees of arc.

### Tumor Xenograft Model and Immunohistochemistry

Five-week-old nude mice were allowed to acclimate for a week before inoculation with A549 cancer cells. Cells (2 × 10^6^) prepared in PBS were injected subcutaneously. After the tumor grew to 50–100 mm^3^, mice were intralesionally injected with harmine (30 mg/kg) every day for 21 days. An equivalent volume of DMSO was injected as a control. Measurements of body weight and tumor dimensions were performed daily. The tumor volume was considered the volume equal to 0.52 × AB^2^, where A is the long axis and B is the short axis.

As described elsewhere [27], solid tumors were removed from sacrificed mice and fixed with 10% formaldehyde. Paraffin-embedded tissues were sectioned at 4 µm and then stained using a Blood Vessel Staining Kit (Millipore). The optical density of stained blood vessels was analyzed by Image-Pro Plus software, according to the following formula: Mean integrated optical density (IOD) = IOD/area of the tumor section.

### Statistical Analysis

Results were statistically analyzed using the Students t-test with Microsoft Excel. Some statistical analyses were performed using two-way analysis of variance followed by the Bonferroni post-hoc test using the GraphPad Quickcalc online web site. All experiments were repeated at least three times. A value of *P*<0.05 was considered significant.

## Results

### Harmine Activates p53 by Inducing Phosphorylation of p53 and Binding to MDM2

**Figure 1:**
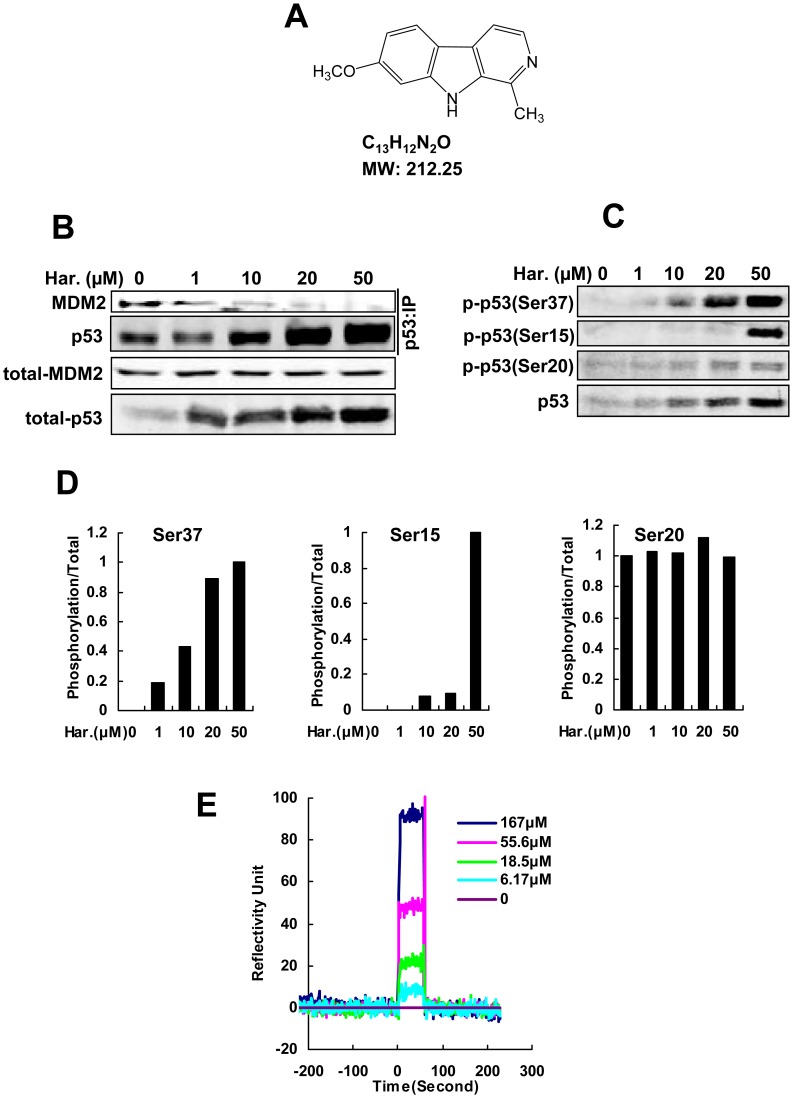
Harmine activates p53 by inducing phosphorylation of p53 and binding to MDM2. (A) Chemical structure, molecular formula and 212.25 molecular weight of harmine. (B) MDM2-p53 interaction assay of harmine-treated HUVECs. After harmine treatment for 48 hours, the endogenous p53-MDM2 interaction was detected by a co-immunoprecipitation assay. (C) HUVECs were treated by harmine for 48 hours. The levels of phosphorylation of p53 at ser-15, ser-20 and ser-37 were assessed by western blot. (D) Quantitative analysis of the phosphorylation level of p53 was performed by Odyssey software. Harmine induced phosphorylation of p53 at ser-15 and ser-37, but not ser-20, in HUVECs. (E) Harmine bound to MDM2 partly, as determined by a ProteOn XPR36 Protein Interaction Array.

A number of reports show that inhibiting the MDM2-p53 interaction can be potentially important for activation of p53 and cancer therapy [3]. We first tested the effect of harmine on the p53-MDM2 interaction in HUVECs using a co-immunoprecipitation assay with anti-p53 and anti-MDM2 antibodies. Our data showed that the amount of MDM2 associated with p53 was dramatically decreased with increasing concentrations of harmine, although endogenous p53 was increased with the addition of harmine (Figure 1B), suggesting that harmine inhibited the endogenous interaction of p53 with MDM2 in a dose-dependent manner.

In the p53 signaling pathway, modifications of p53 phosphorylation at various sites, such as serines 15, 20, and 37, are involved in regulation of p53 functions. p53 phosphorylation deters the association between p53 and MDM2 and prevents p53 ubiquitinization that degrades p53 [28]. To understand the effect of harmine on p53 phosphorylation, we examined three serines sites of p53 by western blot. As shown in Figure 1C and Figure 1D, harmine significantly induced p53 phosphorylation at ser-37 and ser-15 sites in a dose-dependent manner, but had little effect on p53 phosphorylation at ser-20 in endothelial cells. Phosphorylation of p53 occurs in response to some stimulies such as DNA damage. To test the phosphorylation of p53 induced by harmine due to DNA damage, we used γH2AX as a marker for DNA damage and performed comet assay. As shown in Figure S1, harmine did not induce DNA damage obviously. These data suggested that harmine induced p53 phosphorylation maybe not because of DNA damage.

On the other hand, The p53-MDM2 interaction is a pivotal event involved in tumor progression [29], and targeting the MDM2-p53 interaction by small molecules to reactivate p53 has emerged as a promising new strategy for cancer therapy [30]. To determine whether harmine bound to MDM2, we examined the interaction of harmine with MDM2 using a ProteOn XPR36 Protein Interaction Array. Quantitative analysis of the binding data under the deduction of GST showed that harmine rapidly bound to MDM2 protein with a *K*_D_ of 48 µM in an association phase of 70 seconds (Figure 1E and Table 1). Binding of harmine to MDM2 was characterized by a very fast association and dissociation (Figure 1E). This result was fitted by the simplest 1∶1 interaction model (Langmuir model).

**Table 1:** The parameters of the binding of harmine to MDM2.

Parameter	R_max_	R_max_ error	K_D_	K_D_ error	χ^2^
Units	RU	RU	M	M	RU
MDM2	117.93	1.94	4.80E−05	2.07E−06	0.72

### Harmine Induces p53 Stabilization and Nuclear Accumulation

**Figure 2:**
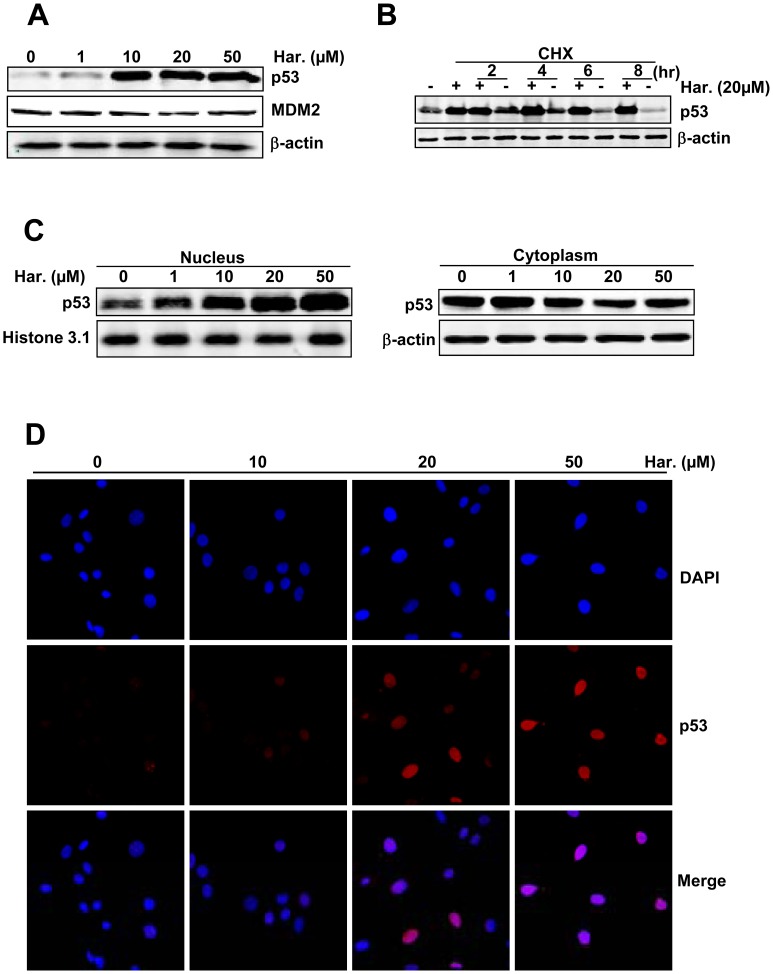
Harmine suppresses the degradation of p53 and increases the accumulation of p53 in the nucleus. (A) Nuclear accumulation of p53 protein and MDM2 expression in HUVECs after harmine treatment for 48 hours. (B) Harmine prevented the degradation of p53 in HUVECs in the presence of CHX. (C) Harmine increased the nuclear levels of p53. Cells were treated with harmine for 48 hours, and then cytoplasmic and nuclear extractions were performed to detect the levels of p53 protein in the cytoplasm and nucleus by western blot. (D) Nuclear accumulation of p53 in the presence of various concentrations of harmine in HUVECs, as determined by immunofluorescence staining with an anti-p53 antibody. Cells were stained with the anti-p53 antibody (red) and nuclei were counterstained with DAPI (blue).

As a negatively regulator of p53, MDM2 promotes p53 degradation [31]. On the other hand, interaction with MDM2 protein also prevents p53 from degradation and promotes p53 stabilization [32]. Therefore, we examined whether expression levels of p53 and MDM2 were affected in harmine-treated cells. Results showed that harmine upregulated p53 protein expression in a dose-dependent manner, but had little effect on the protein expression of MDM2 (Fig. 2A). Next, we examined the stabilization of p53 induced by harmine. As shown in Figure 2B, in the absence of harmine and in the presence of cycloheximide (CHX), which inhibits protein synthesis, the protein level of p53 was lower in cells treated with CHX for 8 hours, compared with that in untreated endothelial cells, indicating p53 degradation during inhibition of protein synthesis. Conversely, in the presence of CHX, the protein level of p53 did not significantly change in harmine-treated endothelial cells. These results suggested that harmine prevented p53 degradation and induced stabilization of p53 in endothelial cells.

A previous report showed that interruption of the p53-MDM2 interaction and p53 phosphorylation results in nuclear accumulation of p53 [33]. To investigate nuclear accumulation of p53 induced by harmine, we examined the expression level of p53 in the nucleus and cytoplasm by western blot using an anti-p53 antibody. As shown in Figure 2C, nuclear levels of p53 were elevated significantly in cells exposed to harmine (left panel), while cytoplasmic levels of p53 hardly changed with the addition of harmine (right panel). To confirm nuclear accumulation of p53 induced by harmine, we examined the expression level of p53 in the nucleus by immunofluorescence staining using the anti-p53 antibody. Our data indicated that harmine increased nuclear localization of p53 in a dose-dependent manner (Figure 2D). These results suggested that p53 stabilization resulted from inhibition of the p53-MDM2 interaction, and p53 phosphorylation induced by harmine was an important factor for nuclear accumulation in endothelial cells.

### Harmine Induces p53 Transcriptional Activity

**Figure 3:**
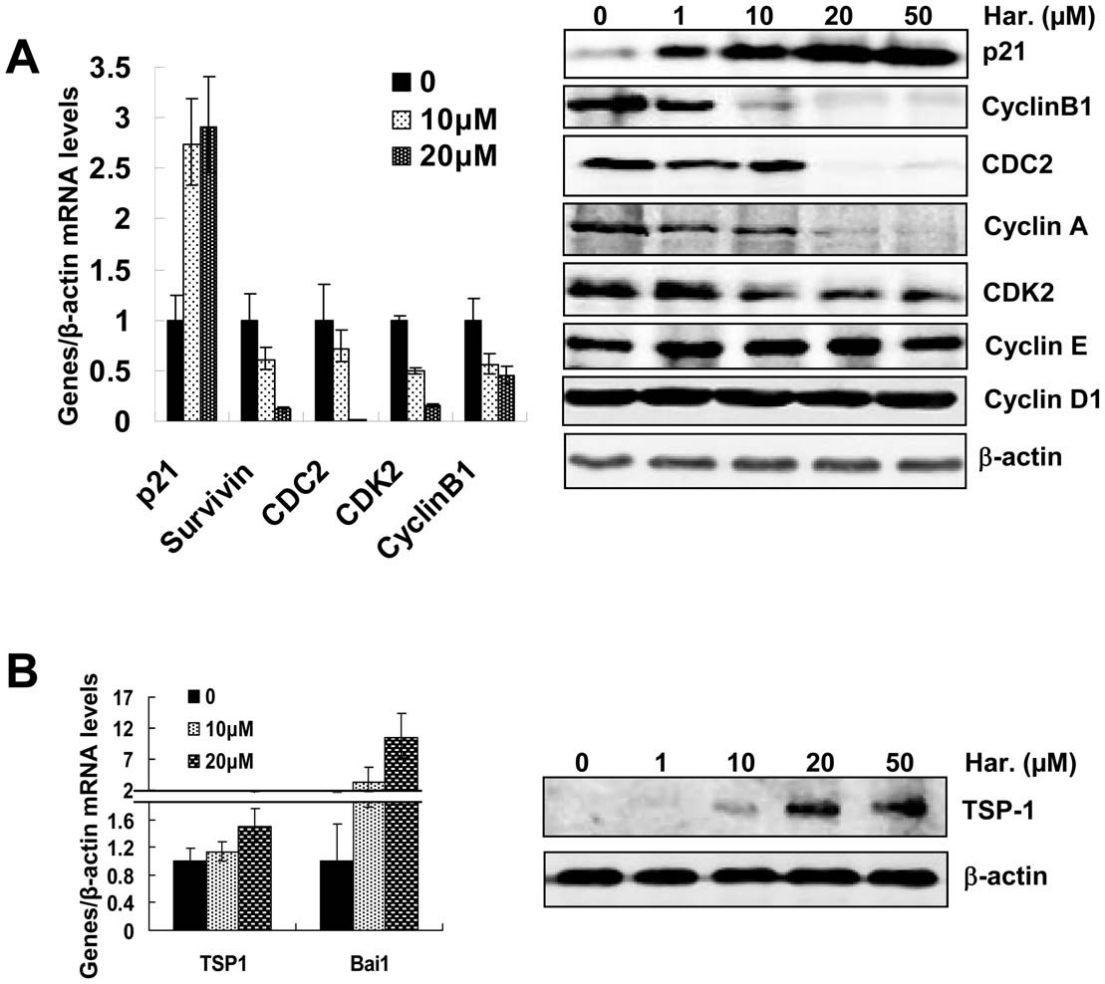
Harmine induces the expression of p53-target genes. (A) Protein expression levels of p21, cyclin B1, CDC2, cyclin A, CDK2, cyclin D1 and cyclin E in HUVECs treated by harmine for 48 hours. (B) Expression levels of endogenous angiogenesis inhibitors TSP-1 and Bai1 in HUVECs. Protein expression level of TSP-1 was detected followed by addition of harmine for 48 hours, the mRNA levels of angiogenesis inhibitors were detected after addition of harmine for 24 hours.

When the p53-MDM2 interaction is disrupted and the protein level of p53 in the nucleus is increased, transcriptional activity of p53 is activated. To further investigate the effect of harmine on the p53-MDM2 signaling pathway, we examined the expression levels of p53-target genes such as *p21, survivin, CDC2, cyclin B1* and *CDK2*. As shown in Figure 3A, left panel, harmine increased the mRNA level of *p21*, and inhibited the expression of *survivin, CDC2, cyclin B1* and *CDK2* in a dose-dependent manner, suggesting that harmine increased p53 nuclear accumulation and activated its transcriptional activity in endothelial cells. Accordingly, harmine inhibited the expression levels of both cyclin B1 and CDC2, which are critical regulators of the G2/M checkpoint [34]. Furthermore, harmine inhibited the expression levels of cyclin A and CDK2, but did not affect the expression of cyclin E and cyclin D1.

To understand the molecular mechanism of harmine in endothelial cells, which are very important in angiogenesis, we examined the expression of downstream p53-target genes, TSP-1 [6] and brain angiogenesis inhibitor 1 (Bai1) [7], which are both endogenous angiogenesis inhibitors. As shown in Figure 3B, harmine increased the mRNA level of *TSP-1* and *Bai1* in a dose-dependent manner in HUVECs and upregulated the expression of TSP-1 protein. These results suggested that harmine activated transcriptional activity of p53, resulting in changes of p53-target gene expression.

### Harmine Induces Apoptosis and Cell Cycle Arrest in HUVECs

**Figure 4:**
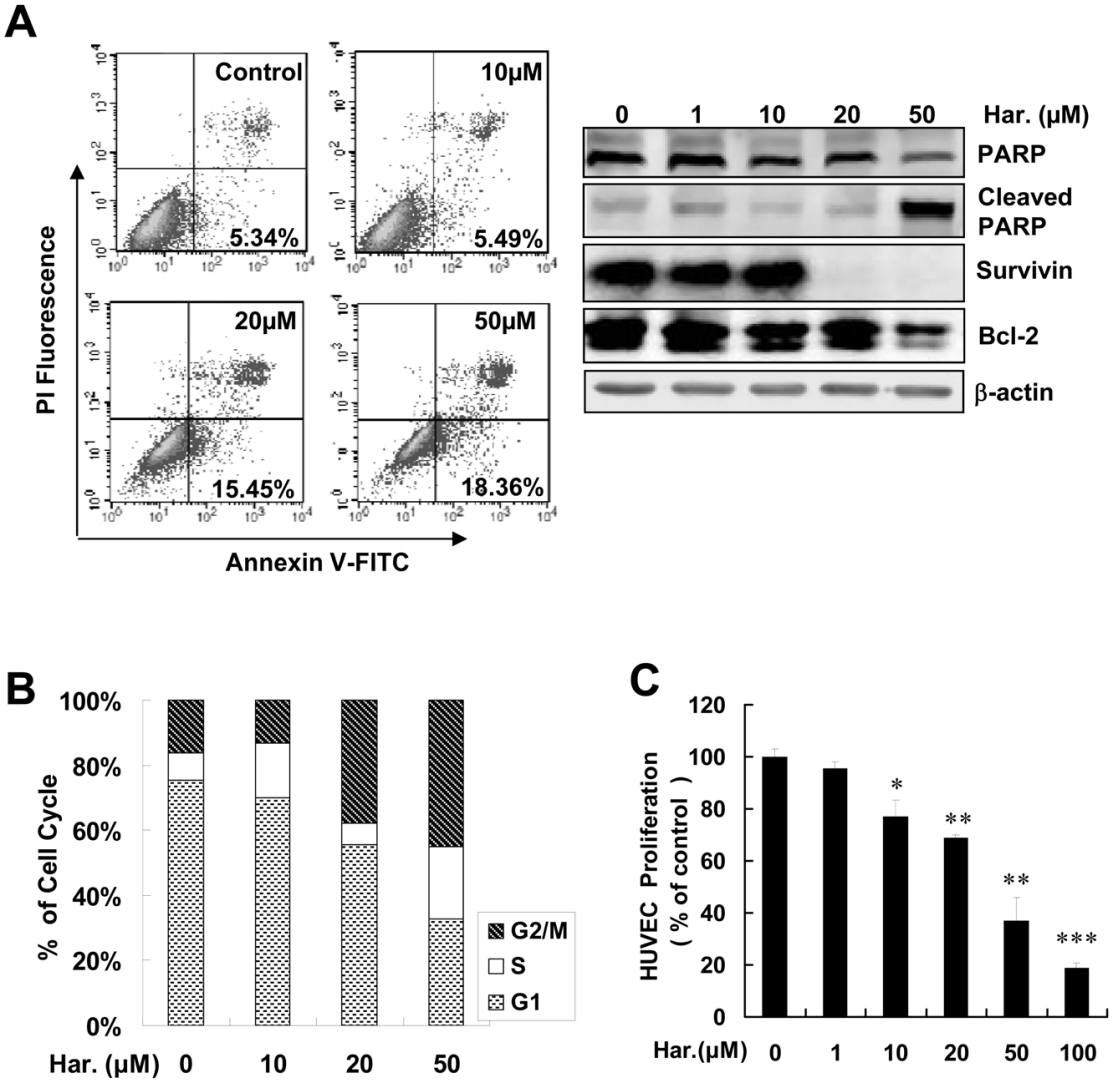
Effects of Harmine on endothelial cell apoptosis , **G2/M arrest and proliferation.** (A) The proportion of apoptotic cells was increased by harmine. Harmine induced cleavage of PARP and suppressed the expression of survivin and Bcl-2. The amount of cleaved PARP and the expression levels of survivin and Bcl-2 in harmine-treated HUVECs were detected by western blot analysis with specific antibodies. (B) Cell cycle arrest of HUVECs by various concentrations of harmine. Cells were treated with various concentrations of harmine for 48 hours, and then the cell cycle was analyzed by flow cytometry. (C) Growth inhibition of HUVECs by harmine, as determined by an MTS assay. All of the experiments were performed followed by addition of harmine for 48 hours. *, *P*<0.05; **, *P*<0.01; ***, *P*<0.001.

As a cellular gatekeeper, activation of p53 induces cell growth arrest and apoptosis [35]. To identify the function of harmine in apoptosis, we performed a cell apoptosis assay using annexin V/PI staining. As shown in Figure 4A, left panel, 20 µM harmine increased HUVEC apoptosis (15.45%) by 3-fold compared with that in untreated cells (5.34%). Harmine-induced HUVEC apoptosis was further confirmed by cleavage of poly (ADP-ribose) polymerase (PARP), a marker of apoptosis (Figure 4A, right panel) [36], and by the decrease of anti-apoptotic proteins Bcl-2 [37] and survivin [38] in endothelial cells (Figure 4A, right panel).

To further identify whether harmine induced apoptosis in p53-depemdent manner, we tried to silence the p53 gene with siRNA targeting p53 mRNA in HUVECs. As shown in Figure S2, 20 µM harmine did not significantly increased HUVEC apoptosis (13.42%) compared with that in untreated cells (11.08%). These results suggested that harmine-induced apoptosis may depend on p53 pathway in HUVECs.

We further examined the effect of harmine on the cell cycle progression of endothelial cells. In the presence of harmine, cells were visibly arrested in G2/M, accompanied by a increased G2/M phase of the cell cycle (Figure 4B and Table 2). To evaluate the effect of harmine on HUVEC proliferation, we performed a cell proliferation assay. Harmine treatment of HUVECs resulted in inhibition of HUVEC proliferation as indicated by a decrease of absorbance at 490 nm, comparing with that of the control (Figure 4C).

**Table 2:** Representation of the percentage in different phases of the endothelial cell cycle distribution by harmine.

Har.(µM) Cell Cycle	0	10	20	50
Sub-G1 (%)	3.65	6.21	6.16	6.27
G1 phase (%)	71.20	63.78	50.03	28.48
S phase (%)	8.20	15.44	5.90	19.30
G2 phase (%)	15.27	12.07	34.21	39.00
Polyploid Population (%)	1.78	2.50	3.70	6.95

### Harmine Inhibits HUVEC Migration and Tube Formation

**Figure 5:**
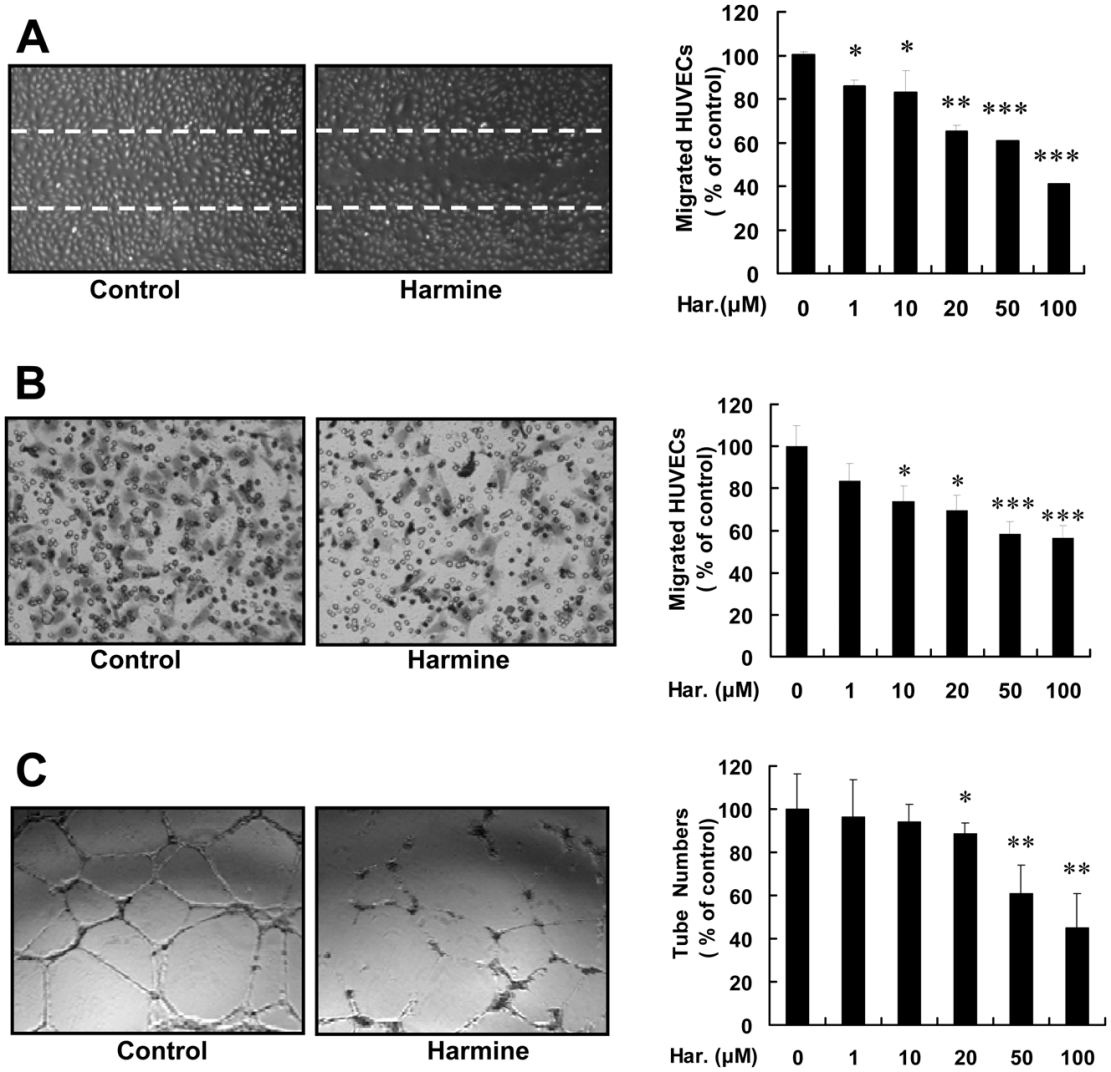
Harmine inhibits HUVEC migration and tube formation. (A) Representative images and quantitative data of a wound healing migration assay in the presence or absence of 20 µM harmine for 8 hours. (B) Results of transwell migration assays of HUVECs treated or untreated by harmine for 8 hours. Right panel indicates quantitative data. (C) Representative images of tube formation assays of HUVECs in the presence or absence of harmine (left panel) for 8 hours, and the quantitative data of tube formation assays (right panel). *, *P*<0.05; **, *P*<0.01; ***, *P*<0.001.

To evaluate the effect of harmine on the migration of HUVECs, we performed wound healing and transwell migration assays. Quantitative analysis by counting the number of migrated cells showed that harmine inhibited cell migration in a dose-dependent manner (Figure 5A and 5B). Endothelial cells are capable of forming capillary-like structures *in vitro*, which mimics the process of capillary formation *in vivo*. Harmine treatment inhibited the ability of HUVECs to organize into capillary-like networks on Matrigel in a dose-dependent manner (Figure 5C). These results revealed that harmine inhibited neovascularization *in vitro*.

### Harmine Inhibits Angiogenesis *ex vivo* and *in vivo*

**Figure 6:**
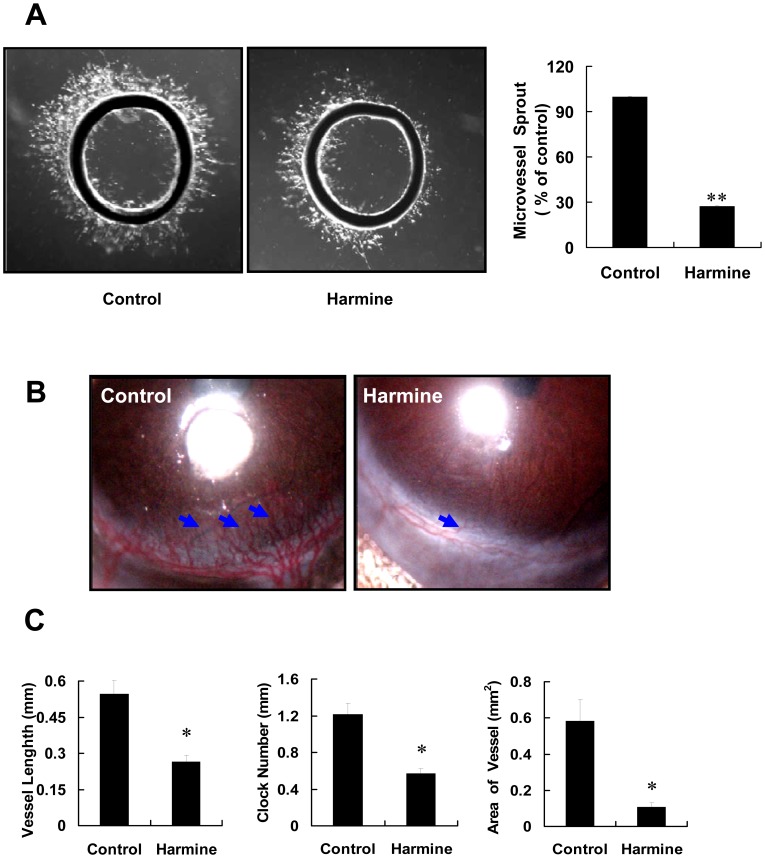
Harmine inhibits angiogenesis *ex vivo* and *in vivo*. (A) Representative images (left panel) and the optical density (right panel) of microvessels sprouting from rat thoracic aorta rings in the absence or presence of harmine. The microvessel density of the untreated group was considered as 100%. (B) Representative images of blood vessels in control eyes (left panel) and harmine-treated eyes (right panel). Black arrows indicate blood vessels. (C) The vessel length, clock number and area of blood vessels in control and harmine-treated groups. At day 7, mice were anesthetized, and images of blood vessels in control and harmine-treated eyes were recorded (n = 10). *, *P*<0.05; **, *P*<0.01.

To assess the anti-angiogenic activity of harmine, a rat thoracic aorta ring assay was performed. As shown in Figure 6A, 50 µM harmine obviously inhibited vessel sprouting from rat aortic rings. The average area of sprouting vessels from thoracic aorta rings in the harmine-treated group was only 30%, compared with that of the control group. To further explore the effect of harmine on angiogenesis *in vivo*, we performed a corneal angiogenesis assay, which is an important animal model to evaluate angiogenesis. Our data showed that 5 µg harmine significantly blocked neovascularization (Figure 6B, arrows) induced by VEGF, as measured by the vessel length, clock number and area of vessels in harmine-treated eyes, compared with those of the control group (Figure 6C). During experimentation, we did not observe symptoms of eye inflammation including keratitis, corneal edema or advanced signs of intraocular inflammation (data not shown). Results of these two assays suggested that harmine inhibited angiogenesis *ex vivo* and *in vivo*.

### Harmine Suppresses Tumor Growth by Inhibiting Tumor Angiogenesis

**Figure 7:**
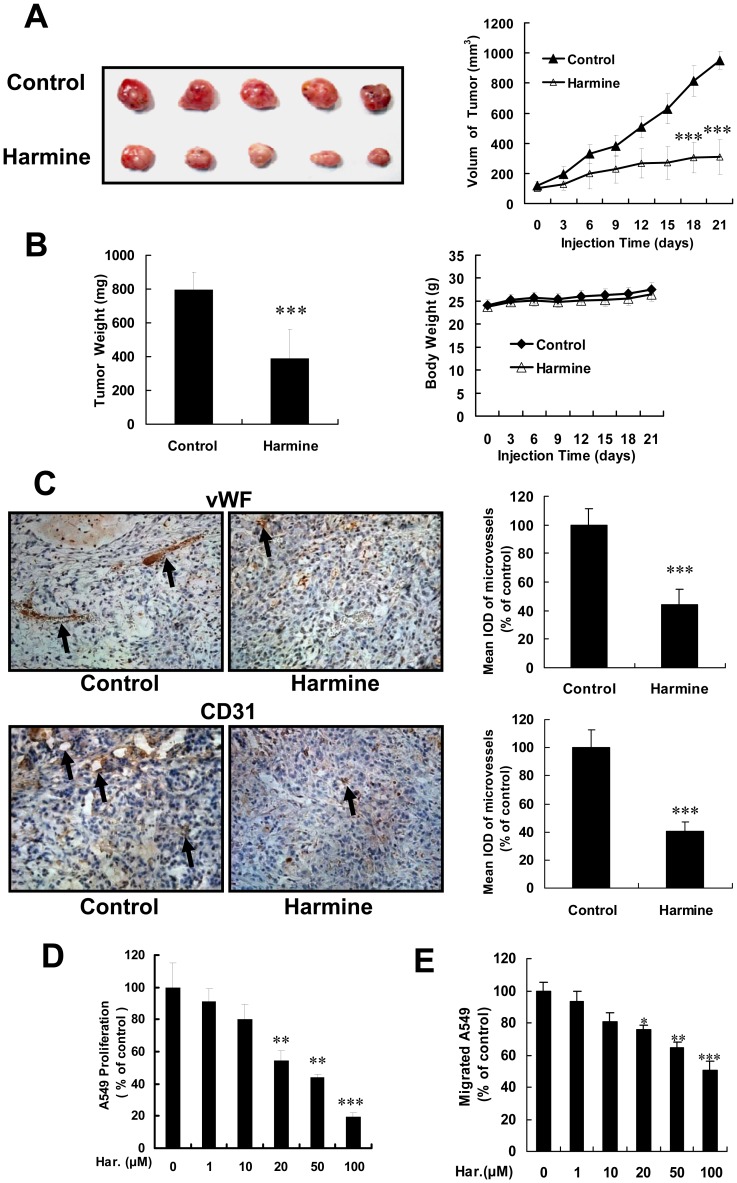
Harmine inhibits tumor growth and angiogenesis. (A) Inhibition of solid tumor growth in harmine-treated xenograft mouse models. Images of solid tumors in control and experimental groups are shown. (B) The size of solid tumors was assessed in the absence or presence of harmine, and statistical results of solid tumors and the body weight of mice. (C) The blood vessels of xenografted tumors in control and harmine-treated groups. Blood vessels were stained using anti-vWF and anti-CD31 antibodies. Black arrows indicate positive blood vessels. (D) Growth inhibition of A549 lung cancer cells treated by harmine for 48 hours, as determined by an MTS assay. (E) The quantitative data of A549 lung cancer cells migration assays. A549 lung cancer cells were treated by harmine for 12 hours after the starvation overnight. *, *P*<0.05;**, *P*<0.01; ***, *P*<0.001.

To determine the effects of harmine on tumor angiogenesis and growth, we performed a xenograft lung tumor assay in nude mice. After treatment for 21 days, 30 mg/kg harmine dramatically inhibited the size (Figure 7A) and weight (Figure 7B, left panel) of tumors, but there was no significant difference in body weight between harmine-treated mice and control mice (Figure 7B, right panel).

To ascertain whether inhibition of solid tumor growth resulted from the decrease of new blood vessels, we examined vessel formation in harmine-treated and control groups by immunohistochemistry using antibodies against endothelial cell markers such as vWF and CD31. Our data indicated that the microvessel density of the harmine-treated group was much less than that of the control group (Figure 7C), suggesting that harmine inhibited lung tumor (A549 cells) growth in a xenograft mouse model by inhibition of angiogenesis.

To further ascertain whether inhibition of angiogenesis is the only reason for the suppression of tumor growth, we examined the effect of harmine on the proliferation and migration of A549 lung cancer cells. Our results showed that harmine also inhibited the proliferation and migration of A549 lung cancer cells (Figure 7D and 7E). These results suggested that harmine inhibited tumor growth by both inhibition of angiogenesis and inhibition of cancer cell proliferation.

## Discussion

**Figure 8:**
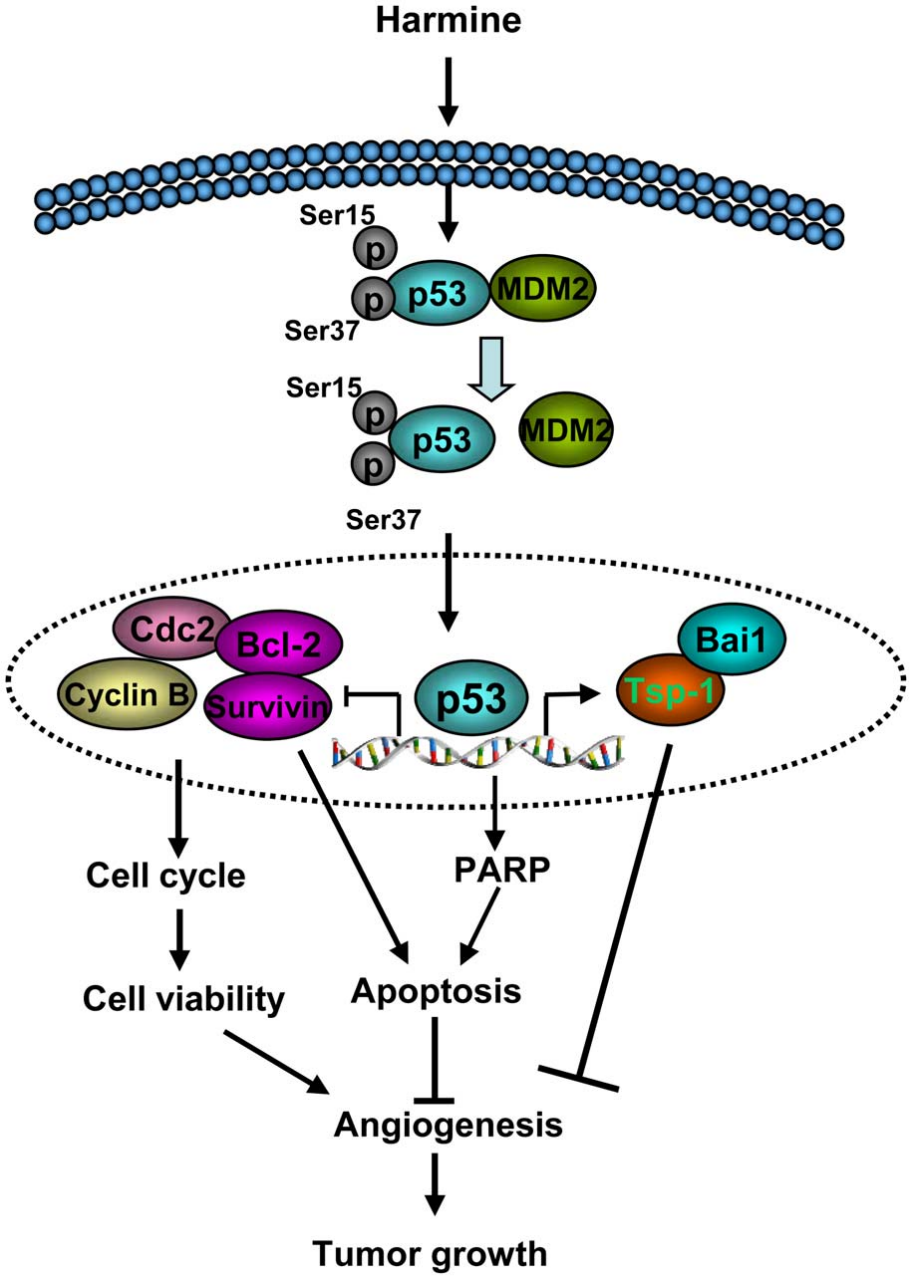
Proposed model for the inhibition of angiogenesis and tumor growth by harmine. Harmine increases the activation of p53 and induces the accumulation of stable p53 in the nucleus. Stable p53 down-regulates its downstream target genes, including *CDC2*, *cyclin B1*, *survivin* and *Bcl-2*, leading to cell cycle arrest and apoptosis of HUVECs. In addition, p53 up-regulates the expression of TSP-1 and Bai1. All these events in endothelial cells result in inhibition of tumor angiogenesis and growth.

Our study as presented here identified non-peptidic carboline harmine as a novel small molecule that possessed an ability to induce p53 level and activity, consequently leading to p53 stabilization, nuclear accumulation and transcriptional activation in endothelial cells. Moreover, harmine not only inhibited angiogenesis *in vitro* and *in vivo,* but also suppressed tumor growth by inhibiting tumor angiogenesis (Figure 8).

In p53 signaling pathway, p53 phosphorylation is one of the key regulatory steps for activating p53 function. In different sites, p53 was phosphorylated by several kinases such as casein kinase II (CK II), DNA-dependent protein kinase (DNA-PK), protein kinase C (PKC) and mitogen-activated protein kinase (MAPK) [39] followed by resulting in nuclear accumulation of p53 [33]. Other study showed that, in response to DNA damage, phosphorylation p53 at serines 15 and 37 by DNA-PK inhibited MDM2 binding to p53 [40]. In addition, phosphorylation of p53 at serine 20 resulted from some other kinases including PIK3 [41] is also thought to play a role in modulating the negative regulation of p53 by MDM2. In this study, we demonstrated that harmine induced p53 phosphorylation at ser-37 and ser-15 sites, but not the ser-20 site (Figure 1C and 1D) followed by activating the p53 signals in endothelial cells (Figure 1B). However, we did not find harmine induced obvious DNA damage (Figure S1) measured by H2AX and comet assays. These results, together with other studies, suggested that harmine induces p53 phosphorylation possibly in response to other factors and further studies in depth will be conducted later.

Several studies have shown that the p53 activation reduces nuclear export of p53, increased transcriptional activity of p53 [42] and leads to growth arrest or apoptosis [43]. We found that harmine induced p53 stabilization (Figure 2A and 2B) and nuclear accumulation (Figure 2C and 2D). Furthermore, we also found that harmine regulated the expression of downstream p53-target genes, including *cyclin B1*, *cyclin A* and *CDC2* (Figure 3A), which are involved in the endothelial cell cycle. On the other hand,importantly, major roles of the p53 protein in cancer had also been shown to limit angiogenesis by some mechanisms [5] including directly increasing the production of endogenous angiogenesis inhibitors and potential angiogenic markers such as Bai1 and TSP-1 [44].

Our present study demonstrated that, in the dose-dependent manner, harmine induced the expression of Bai1 and TSP-1 (Figure 3B). These results, together with the results shown in Figure 1, suggested that harmine induced nuclear accumulation of p53 and activated p53 transcriptional activities due to reactivation of p53.

Angiogenesis is necessary of tumor growth. In this study, using a xenograft mouse model and immunohistochemistry with anti-vWF and anti-CD31 antibodies (Figure 7), we demonstrated that harmine inhibited tumor growth with low side-effects by suppressing tumor angiogenesis. A recent report [45] showed the effect of harmine on angiogenesis *in vitro* and tumor-specific capillary formation, but it did not demonstrate the anti-tumor activity of harmine by suppressing tumor angiogenesis. In the present study, we demonstrated that, under the effective dosage, inhibiting tumor angiogenesis may be the important aspect of antitumor activity of harmine and we expounded the novel roles of harmine in anti-tumor activity by inhibiting angiogenesis.

Some other studies showed that p53 induced the activity of PARP [46] and inhibited survival by activating p21, a regulator of apoptosis and the cell cycle [47]. In addition,VEGF/VEGFR2 independent control of the anti-apoptotic pathway in endothelial cell is mediated by the activation of p53/caspases [48]. So, VEGF and p53 expression are significantly correlated and might be associated with relevant events involved in tumor biology [49]. In this study, we demonstrated that harmine induced endothelial cell apoptosis (Figure 4A), cell cycle arrest (Figure 4B) and reduced cell viability (Figure 4C) mainly depending on p53 pathways. Furthermore, harmine not only inhibited angiogenesis *ex vivo* in an aorta ring assay (Figure 6A), which is consistent with a recent report [45], but also inhibited VEGF-induced angiogenesis *in vivo* in a mouse corneal assay (Figure 6B and 6C), the gold standard of angiogenesis analysis [21]. All these results suggest that harmine inhibits angiogenesis *in vitro, ex vivo* and *in vivo*.

In conclusion, our results not only indicate that harmine is a novel activator of p53 in endothelial cells (Figure 8), but also suggest that harmine may exert its biological activities on other angiogenic diseases through p53 signaling pathway.

## Supporting Information

Figure S1 **Hrmine did not induce DNA damage.** (A) Immunofluorescent foci formation of γH2AX in HUVECs in the presence or absence of harmine for 48 hours. (B) The quantitative data of γH2AX foci in HUVECs treated by various concentrations of harmine. At least 100 cells were measured per sample. (C) HUVECs were treated by various concentrations of harmine for 48 hours, and then alkaline comet assay was performed. At least 500 cells were counted per sample.
(PPT)

Figure S2 **Harmine did not have significant effect on aopotosis of HUVEC transfected with p53 siRNA.** (A) The mRNA levels of p53 in HUVECs after the p53 siRNA interference for 36 hours. HUVECs were transfected with p53 siRNA at the concentration of 50 nM and 100 nM. The effect of these concentrations was similar. (B) The expression of p53 protein was detected by western blot in HUVECs transfected with p53 siRNA for 72 hours at the concentration of 50 nM and 100 nM (left panel) and the quantitative data of p53 expression (right panel). 100 nM p53 siRNA inhibited p53 expression (48.2%) compared with the negative control. (C) The proportion of apoptotic cells induced by harmine in HUVECs transfected with p53 siRNA(100 nm). After the transfection for 16 hours, HUVECs were treated by various concentrations of harmine for 48 hours.
(PPT)
