# Supplementary figures and images for: A Natural Small Molecule Harmine Inhibits Angiogenesis and Suppresses Tumour Growth through Activation of p53 in Endothelial Cells
Source: PLoS One. 2012 Dec 27;7(12):e52162. doi: 10.1371/journal.pone.0052162 (PMC3531399; doi:10.1371/journal.pone.0052162)

## Slide 1
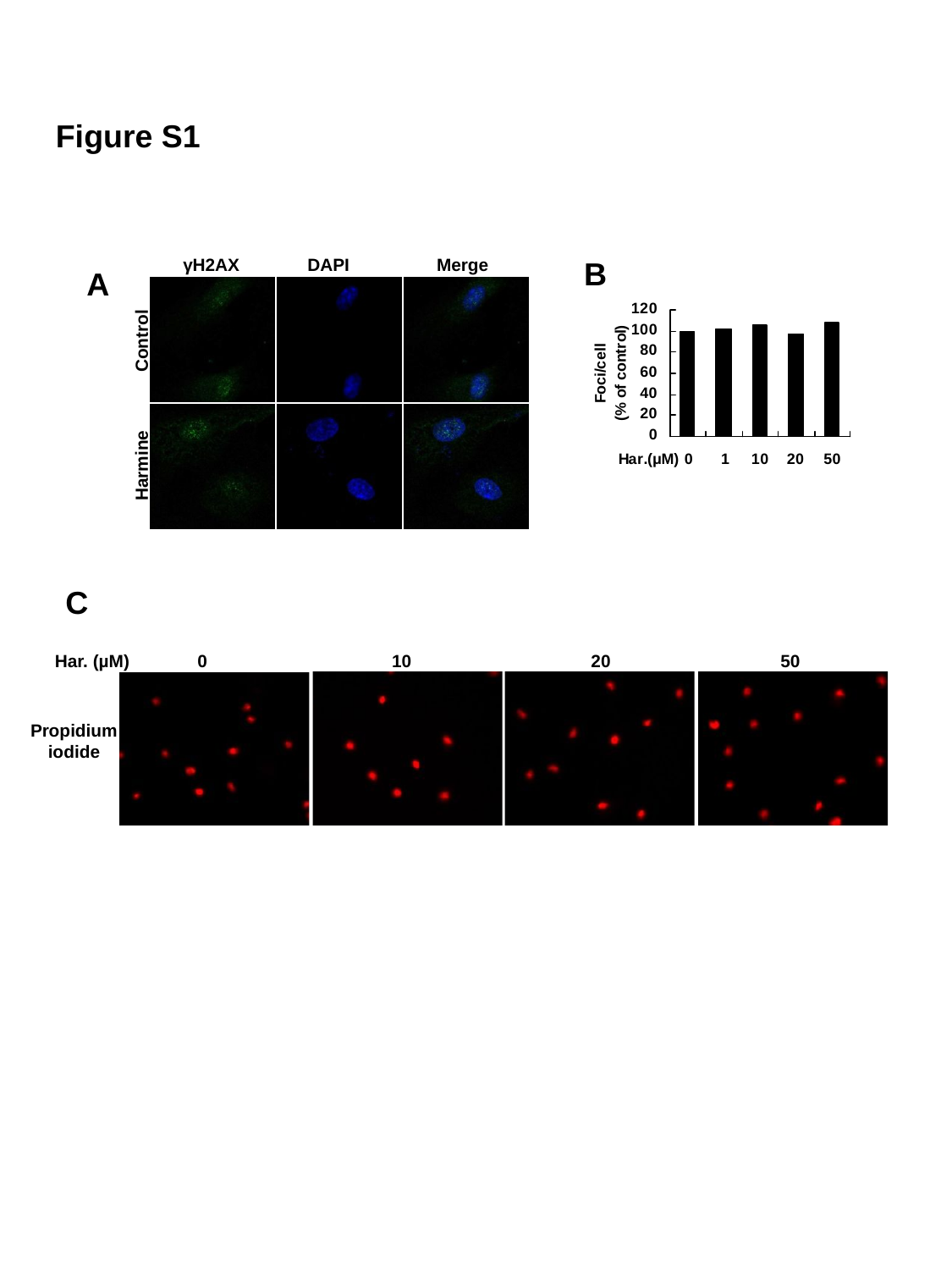

Figure S1
γH2AX DAPI Merge
A
Harmine Control
B
C
Har. (µM) 0 10 20 50
Propidium
 iodide

Supplement: Figure S1 — Hrmine did not induce DNA damage. (A) Immunofluorescent foci formation of γH2AX in HUVECs in the presence or absence of harmine for 48 hours. (B) The quantitative data of γH2AX foci in HUVECs treated by various concentrations of harmine. At least 100 cells were measured per sample. (C) HUVECs were treated by various concentrations of harmine for 48 hours, and then alkaline comet assay was performed. At least 500 cells were counted per sample. (PPT) [file pone.0052162.s001.ppt]
