# Supplementary material for: A Natural Small Molecule Harmine Inhibits Angiogenesis and Suppresses Tumour Growth through Activation of p53 in Endothelial Cells
Source: PLoS One. 2012 Dec 27;7(12):e52162. doi: 10.1371/journal.pone.0052162 (PMC3531399; doi:10.1371/journal.pone.0052162)
Supplement: Figure S2 — Harmine did not have significant effect on aopotosis of HUVEC transfected with p53 siRNA. (A) The mRNA levels of p53 in HUVECs after the p53 siRNA interference for 36 hours. HUVECs were transfected with p53 siRNA at the concentration of 50 nM and 100 nM. The effect of these concentrations was similar. (B) The expression of p53 protein was detected by western blot in HUVECs transfected with p53 siRNA for 72 hours at the concentration of 50 nM and 100 nM (left panel) and the quantitative data of p53 expression (right panel). 100 nM p53 siRNA inhibited p53 expression (48.2%) compared with the negative control. (C) The proportion of apoptotic cells induced by harmine in HUVECs transfected with p53 siRNA(100 nm). After the transfection for 16 hours, HUVECs were treated by various concentrations of harmine for 48 hours. (PPT) [file pone.0052162.s002.ppt]

## Slide 1
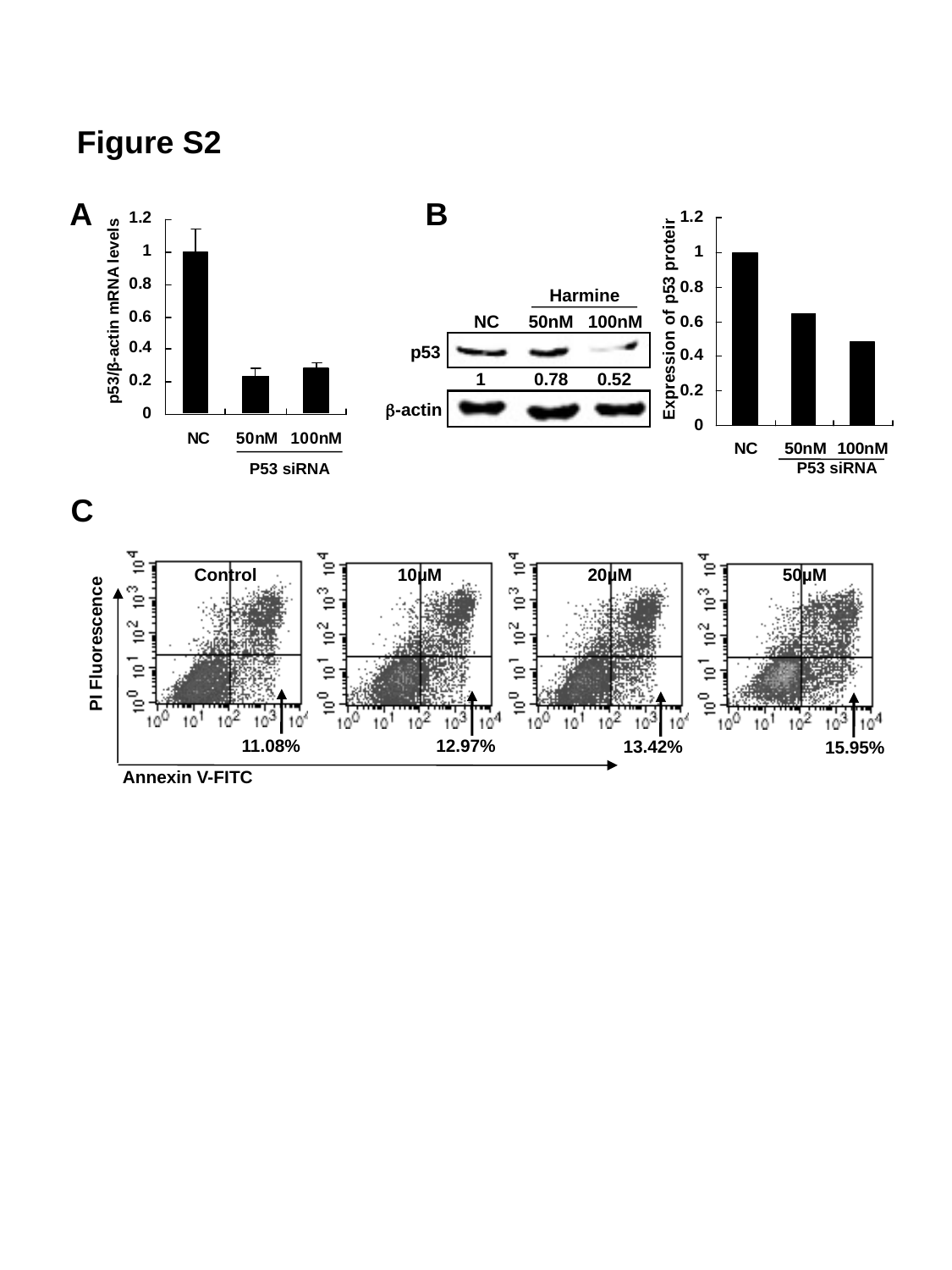

Figure S2
A
p53/β-actin mRNA levels
P53 siRNA
B
Harmine
NC 50nM 100nM
p53
1 0.78 0.52
β-actin
P53 siRNA
C
 Control 10µM 20µM 50µM
PI Fluorescence
11.08%
12.97%
13.42%
15.95%
Annexin V-FITC
